# Lidocaine‐induced systemic toxicity complicating radiofrequency ablation of benign thyroid nodule procedure: A case report and review of literature

**DOI:** 10.1002/ccr3.4910

**Published:** 2021-10-10

**Authors:** Van Bang Nguyen, Van Vy Hau Nguyen, Linh Pham Nguyen Tuyen, Chi Van Le

**Affiliations:** ^1^ Center of Endocrinology and Diabetes Family Hospital Da Nang Vietnam; ^2^ Department of Internal Medicine Hue University of Medicine and Pharmacy Hue University Hue City Vietnam

**Keywords:** case report, lidocaine‐induced systemic toxicity, radiofrequency ablation, thyroid nodules

## Abstract

The systemic toxicity of lidocaine is an extremely rare complication of thyroid RFA procedure, and it can be life‐threatening. The quick recognization of its symptoms and intravenous use of lipid emulsion are essential to preventing mortality.

## Graphical abstract

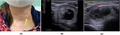

## 1 INTRODUCTION

This study describes a patient with lidocaine‐induced systemic toxicity during radiofrequency ablation of benign thyroid nodule procedure. The quick symptom recognization and intravenous lipid emulsion treatment are cornerstone to prevent mortality. This report emphasizes and discusses an extremely rare complication of thyroid RFA procedure.

In recent years, radiofrequency ablation (RFA) has been emerging as one of the treatments of thyroid nodule(s). Theoretically, the RFA procedure usually used 2 basic techniques including the moving‐shot technique and the trans‐isthmic approach with the guidance of ultrasonography (US) after local anesthesia with 2% lidocaine at the needle‐puncture site and thyroid capsule.^1^, ^2^ To relieve pain, this procedure required a large amount of 2% lidocaine which could reach 25 ml in volume.^3^

Systemic toxicity from using lidocaine, particularly in RFA procedure, is extremely scarce, but can reduce the effectiveness of treatment and even be potentially life‐threatening due to its toxic manifestations to the central nervous system (CNS) and cardiovascular system (CVS).^3^, ^4^, ^5^ To the best of our knowledge, there are few reports of lidocaine‐induced systemic toxicity during thyroid RFA procedure in the literature. This report aims to describe a case of lidocaine‐induced systemic toxicity in a healthy 48‐year‐old female who underwent an RFA procedure for benign thyroid nodule at an outpatient clinic and to raise awareness among endocrinologists/surgeons/radiologists using RFA for the treatment of thyroid nodule(s) to identify and manage patients with lidocaine toxicity.

## 2 CASE PRESENTATION

A 48‐year‐old South African woman presented with a left neck mass for 1 year with cosmetic concerns and no compressive symptoms. She reported no significant history of cardiovascular disease, and no history of food or drug allergies. No family history of any thyroid diseases and any allergies was reported. She was admitted to the hospital for a tentative thyroid radiofrequency ablation.

**FIGURE 1:**
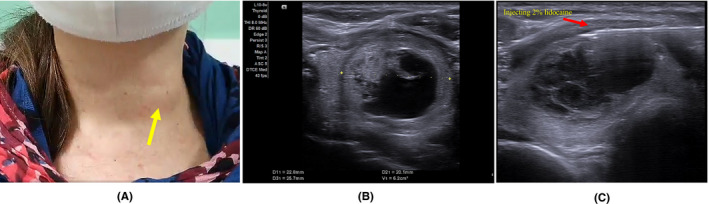
A 48‐year‐old South African woman presented with a left neck mass. (A) A soft mass in the left thyroid lobe, which was moving on swallowing (yellow arrow). (B) A single, mixed echogenicity mass on the left thyroid lobe, with no calcification, no vascularity, and no lymph node involvement. (C) Injecting 10ml of 2% lidocaine at the peri‐thyroidal area under ultrasound guidance (red arrow)

Her body mass index was 24.5 kg/m^2^ (weight 66.7 kg; height 165 cm), and her blood pressure, heartbeat, SpO2, and body temperature were normal (113/71 mmHg, 70 beats/min, 96%, and 37.2°C, respectively). On physical examination, there was a soft mass in the left thyroid lobe, which was moving on swallowing (Figure 1A). Her report was no compressive symptoms, with a symptom score of 5/10 on a visual analog scale and a cosmetic score of ¾. The physical examination of heart, lung, abdomen, and CNS was normal.

Before the procedure, thyroid function tests (FT4, thyrotropin) and other tests (CBC, liver, and renal function tests, prothrombin time) were normal with serum FT4 1.16 ng/dl (normal range [NR] 0.93–2.22); thyrotropin 4.17 microUI/ml (NR 0.27–4.2); prothrombin time 111%, and INR 1.0. Her chest X‐ray showed no abnormal findings. ECG showed normal sinus rhythm at a rate of 73 bpm. The thyroid ultrasonography showed a single, mixed echogenicity mass (cystic part of 50%) on the left thyroid lobe, with no calcification, no vascularity, and no lymph node involvement (Figure 1B). Nodule volume was 6.2 ml (W × H × D: 22.8 × 20.1 × 25.7 mm). 2 separate FNAs of the thyroid nodule revealed a colloid nodular hyperplasia with hyperplastic thyrocytes and fluid colloid (Bethesda II) affirmed the diagnosis of the large benign thyroid nodule. Because of her fear of surgery complications, we proposed RFA of the nodule.

In a preliminary assessment, a procedure safety checklist (medical conditions, drugs using, allergies) and written informed consent for the procedure (efficacy, possible complications) were obtained. The emergency drug box (including methylprednisolone, epinephrine, diphenhydramine, and lipofundin 20% (lipid emulsion)) and the vital signs monitor machine were routinely prepared. The patient underwent an intravenous infusion in 500 ml of 0.9% saline during and after thyroid RFA. In the RFA procedure, after finishing skin sterilization and careful local anesthesia with 10 ml of 2% lidocaine at the needle‐puncture site and peri‐thyroidal area under ultrasound guidance (Figure 1C), complete fluid aspiration was performed before ablating the nodule and its vascularity via the trans‐isthmic approach and the moving‐shot technique.

After 15 min of ablation, the patient started feeling circumoral numbness, dizziness, nausea, and blurred vision. Her skin became cold, and no rash appeared. Shortly, she was hypotensive, bradycardia, and hypoxia with a blood pressure of 80/60 mmHg, a heartbeat of 34 bpm, and SpO2 of 74%. The ablation procedure was aborted immediately. Emergency management was rapidly started including “call help” with red code, nasal cannula oxygen therapy to maintain SpO2 of 100%, monitor the cardiovascular system, and support the patient with keeping rapid intravenous fluids flow (0.9% saline). At the same time, we administrated of lipid emulsion therapy (lipofundin 20%) in different intravenous way: first, intravenous bolus 1.5 ml/kg over 1–2 min (100 ml) and then intravenous infusion 0.25 ml/kg/min (~17 ml/min). The normal sinus rhythm and blood pressure were restored within 5 min of intravenous lipid injection with 60 bpm and 110/80 mmHg. Her mental status became stable. The total used volume of lipofundin 20% was 500 ml. The patient was subsequently transferred to our intensive care unit for follow‐up and supportive treatment. She was discharged 12 h later.

## 3 DISCUSSION AND CONCLUSIONS

RFA has been worldwide used for thyroid nodules treatment with high feasibility of performing in an outpatient room.^6^, ^7^ For pain relief while ablating procedure, this therapy requires local anesthesia by injecting a high volume of lidocaine 1% or 2% lidocaine at the needle‐puncture site and peri‐thyroidal area under ultrasound guidance.

Lidocaine, which has been used as a local anesthetic medication in almost all medical specialties since its advent in 1948, causes transient blockade of sensory and motor when being injected in closeness to neural tissue.^8^, ^9^ In RFA for thyroid nodules, lidocaine is recommended using from the Korean Society of Thyroid Radiology.^1^, ^10^ Its mechanism is to close voltage‐gated Na^+^ channels inside the cell, inhibiting the next channel activation and interrupting the large transient Na^+^ flow associated with membrane depolarization.^11^, ^12^ The total rate of lidocaine‐induced systemic toxicity ranges from 1:10,000 (epidurals anesthesia) to 1:2000 (peripheral nerve blocks).^4^ Because electrophysiological changes affect earlier CNS than the CVS, neurological symptoms including dizziness, tinnitus, and perioral numbness usually manifest.^4^, ^13^

Physicians need to limit the total dose given as low as possible to minimize its systemic toxicity. Lidocaine toxicity was well correlated with the total dose (usually 4.5 mg/kg) and the rate of absorption, which depends on the blood supply of that tissue.^14^, ^15^, ^16^ However, lidocaine toxicity may occur even at a lower dose in case of alpha‐1‐acid glycoprotein and albumin deficiencies.

It has been discussed in literature that circumoral numbness, tongue paresthesia, and dizziness are early symptoms. Blurred vision and tinnitus may be sensory manifestations. The next neurological symptoms include nervousness, restlessness, agitation, and paranoia, which may develop into seizures. Unconsciousness and coma can occur if lidocaine is largely overdosed. Adverse effects of relative lidocaine overdoses are hypotension and bradycardia which sometimes happen during local anesthesia near the CNS.^14^, ^15^ Thus, the diagnosis of lidocaine‐induced systemic toxicity is based on clinical symptoms. Considering this complication is based on important factors such as the site of injection, dose, and the timing. It is important to notice that even low doses may cause CNS toxicity if injected directly into an artery accidentally.

Under ultrasound guidance, lidocaine was injected at the needle‐puncture site and peri‐thyroidal area for pain relief during RFA procedure. Having a rich blood supply and locating near CNS, the thyroid gland increases the rate of lidocaine absorption and toxicity.^17^ In our case, the total amount of lidocaine used for this patient of 66.7 kg accounted for only 10 ml 2% lidocaine (200 mg) during the procedure, which could not be considered an overdose with respect to the weight (2.99 mg/kg).

Other factors leading to hypotension and bradycardia in our patient were the thermal injuries of the vagus nerve and anaphylaxis. Our patient had the early symptoms of lidocaine toxicity such as circumoral numbness, dizziness, nausea, and blurred vision before developing hypotension and bradycardia. Thus, heat‐induced injury of the vagus nerve can be excluded. Allergic reactions to lidocaine are extremely rare compared to the ester‐type local anesthetics, because of its class of the local amide anesthetics.^16^ Our patient fully recovered after administrating lipid emulsion therapy (lipofundin 20%) and was discharged after 12 h.^18^, ^19^

Reviewing cases with lidocaine toxicity in radiofrequency ablation of thyroid nodule procedure in the literature, keywords including “lidocaine toxicity”, “thyroid”, “radiofrequency ablation”, “case report” were used to search for reported cases on Google Scholar, only one case was reported by Cherry Kim and her colleagues (its percentage of 0.1%). In Kim's report, lidocaine toxicity developed immediately post‐ablation with transient mild mental confusion and was attributed to overdose with respect to the weight. The patient fully recovered within 20 min.^3^

From our case and literature review, to avoid lidocaine toxicity in RFA of thyroid nodule, first, physicians must obtain written informed consent and procedure safety checklist (medical conditions, drugs using, allergies). Second, doctors document the amount of lidocaine used during the RFA procedure or can combine epinephrine with lidocaine to prolong nerve block and increase the maximum dose.^16^ Third, any changes in neurologic signs or symptoms should be considered as a possible manifestation of lidocaine toxicity in the procedure. Finally, lipid emulsion is one of the important drug in the emergency drug box.^14^, ^15^, ^18^, ^19^

In conclusion, the systemic toxicity of lidocaine is one of the thyroid RFA procedure complications and it can be life‐threatening. The quick recognization of its symptoms and intravenous use of lipid emulsion are essential to preventing mortality.

## CONFLICTS OF INTEREST

Conflict of interest relevant to this article was not reported.

## AUTHOR CONTRIBUTIONS

All authors contributed to data analysis, drafting, or revising the article, have agreed on the journal to which the article will be submitted, gave final approval of the version to be published, and agree to be accountable for all aspects of the work.

## CONSENT

Written informed consent form was given to patient.

## Data Availability

Availability of data and materials supporting our findings will be shared upon request.
